# Supplementary material for: Impact of two endotracheal tube fixation on the incidence of peri-oral lesions: Elastic adhesive strips versus cord in a protective sheath. Study protocol for a cluster cross-over randomized trial
Source: PLoS One. 2024 Feb 8;19(2):e0297349. doi: 10.1371/journal.pone.0297349 (PMC10852271; doi:10.1371/journal.pone.0297349)
Supplement: S1 Protocol — (PDF) [file pone.0297349.s002.pdf]

FIXATUB protocol

RCB Registration No.: 2020-A00461-38
DGOS NUMBER : PHRIP-19-0140
Ref : CHD 032-20

"Comparison of two methods of Fixation of endotracheal tube in the ICU: elastic adhesive strips vs. cord in a protective sheath. A randomized, multicenter, cluster and crossover controlled study. FIXATUB" STUDY

Coordinator:

ZINZONI VANESSA
Departemental Hospital Center - Vendee
Clinical Research Unit

Methodologist:

Lucie PLANCHE
Vendée Departemental Hospital Center
Clinical Research Unit

Sponsor:

Centre hospitalier Départemental Vendée
Clinical Research Unit
Boulevard Stéphane MOREAU
85 925 LA ROCHE SUR YON Cedex 09
Tel : 02 51 44 65 72
Fax : 02 51 44 65 85

SIGNATURE PAGE

SIGNATURE OF THE SPONSOR

<table>
<tr><td colspan="3">The sponsor agrees to conduct this study in accordance with all applicable laws and regulations that may apply to the research and in accordance with the protocol.</td></tr>
<tr><td>Name and position of signing official :
For the Sponsor and by delegation of the Director General, the Director
of Medical Affairs and Research</td><td>Date:</td><td>Signature:</td></tr>
</table>

SIGNATURE OF THE INVESTIGATORS

<table>
<tr><td colspan="4">I have read all the pages of the protocol for the clinical trial sponsored by the CHD Vendée. I confirm that it contains all the information necessary for the conduct of the trial. I undertake to carry out the trial in compliance with the protocol and the terms and conditions defined therein. I agree to conduct the trial in compliance with :

- The principles of the "Declaration of Helsinki",
- International (ICH - International Conference of Harmonization) and French (NF ISO 14155) rules and recommendations of good clinical practice
- European regulations and/or national legal and regulatory provisions relating to clinical investigations.
- The legislative and regulatory provisions of the Public Health Code applicable to RIPHs category 2 as well as the associated application texts

I also agree that the investigators and other qualified members of my team will have access to this protocol and to the documents related to the conduct of the trial to enable them to work within the provisions of these documents.</td></tr>
<tr><td>Coordinating Investigator</td><td>Name:</td><td>Date:</td><td>Signature:</td></tr>
<tr><td>Principal Investigator</td><td>Name and Institution:</td><td>Date:</td><td>Signature:</td></tr>
</table>

LIST OF ABBREVIATIONS

ANSM	National Agency for the Safety of Medecines and Health Products
AMM	Marketing Authorization
ARC	Clinical Research Associate (monitor)
BPC	Good Clinical Practice
ISC	Independent Safety Committee
CPP	Committee for the Protection of Persons
CNIL	National Commission for Information Technology and Civil Liberties
CRF	Case Report Form
eCRF	Electronic Case Report Form
EMA	European Medicines Agency
	Serious Adverse Event
ETT	Endotracheal tube
SAE	Serious Adverse Event
ICH	International Conference on Harmonization
MD	Medical Device
MR	CNIL Reference Methodology
NPUAP	National Pressure Ulcer Advisory Panel
PVC	Polyvinyl chloride
SPC	Summary of Product Characteristics
MDR	MD Regulation
ROMPIS	Reaper Oral Mucosa Pressure Injury Scale
SIP	Situation of special interest
TEC	Clinical Study Technician
VM	Mechanical ventilation

TABLE OF CONTENTS

SIGNATURE PAGE 2

LIST OF ABBREVIATIONS 3

TABLE OF CONTENTS 4

INTRODUCTION 6

1. JUSTIFICATION OF THE STUDY 7
   - 1.1. POSITIONING OF THE RESEARCH 7
   - 1.2. BENEFITS AND RISKS TO INDIVIDUALS INVOLVED IN RESEARCH 10

2. OBJECTIVES AND EVALUATION CRITERIA.... 13
   - 2.1. OBJECTIVE AND PRIMARY ENDPOINT 13
   - 2.2. OBJECTIVES AND SECONDARY ENDPOINTS 13

3. STUDY POPULATION 15
   - 3.1. DESCRIPTION OF THE POPULATION 15
   - 3.2. INCLUSION CRITERIA 15
   - 3.3. CRITERIA FOR NON-INCLUSION 16

4. DESIGN AND CONDUCT OF THE STUDY 17
   - 4.1. GENERAL METHODOLOGY OF THE RESEARCH 17
   - 4.2. SCHEME OF THE STUDY 18
   - 4.3. SCHEDULE OF THE STUDY 19
   - 4.4. DESCRIPTION AND JUSTIFICATION OF THE INTERVENTION STUDIED 25
   - 4.5. DESCRIPTION OF THE EVALUATION AND THE DATA COLLECTED 26
   - 4.6. IDENTIFICATION OF ALL SOURCE DATA NOT INCLUDED IN THE MEDICAL RECORD 28
   - 4.7. RULES FOR TERMINATION OF A PERSON'S PARTICIPATION 28

5. SAFETY ASSESSMENT 30
   - 5.1. DEFINITIONS 30
   - 5.2. PROTOCOL SPECIFICS 32
   - 5.3. ROLE OF THE INVESTIGATOR 35
   - 5.4. ROLE OF THE SPONSOR 41
   - 5.5. CONTACT VIGILANCE 43
   - 5.6. INDEPENDENT SUPERVISORY COMMITTEE (ISC) 44

6. DATA MANAGEMENT AND STATISTICS 46
   - 6.1. COLLECTION AND PROCESSING OF STUDY DATA 46
   - 6.2. STATISTICS 47

7. ADMINISTRATIVE AND REGULATORY ASPECTS 52
   - 7.1. RIGHT OF ACCESS TO SOURCE DATA AND DOCUMENTS 52
   - 7.2. DATA PRIVACY 52
   - 7.3. MONITORING OF THE TRIAL 53
   - 7.4. INSPECTION / AUDIT 53
   - 7.5. DECLARATION TO THE COMPETENT AUTHORITIES 53
   - 7.6. AMENDMENTS TO THE PROTOCOL 54
   - 7.7. COMPUTERIZED DATA AND SUBMISSION TO THE CNIL 54
   - 7.8. PATIENT INFORMATION 54
   - 7.9. FINANCING AND INSURANCE 56
   - 7.10. RULES FOR PUBLICATION 56
   - 7.11. ARCHIVING OF SOURCE DATA 57

8. REFERENCES.. 58

INTRODUCTION

Each year in France, nearly 100,000 patients hospitalized in intensive care unit (ICU) for more than 48 hours require invasive respiratory assistance with the placement of an endotracheal tube (ETT) introduced most often by the oro-tracheal route.

Checking the fixation of the ETT is a multi-daily nursing procedure in the ICU.
Proper tube fixation is essential to ensure effective ventilation while minimizing potential complications such as accidental extubations. However, the fixation system chosen can lead to peri-oral injuries such as pressure sores, shearing or mucocutaneous tearing. These lesions are painful for patients and often unsightly.

There are no formal recommendations for the use of a particular fixation system. Thus, the systems used to fix the endotracheal tube vary from one service toanother.

In a survey we conducted in early 2019 among 29 intensive care units in France, it appears that the most commonly used fixation systems are the adhesive elastic band and the fixation cord protected by a sheath.

In order to compare these 2 systems, we propose to conduct the Fixatub study, a prospective multicenter study with cluster and cross-over randomization.

The objective of this study is to demonstrate that the elastic adhesive tape fixation strategy decreases the risk of developing a peri-oral lesion during the time the orally inserted endotracheal tube is held in place.

This will be the first multicenter randomized study on this topic. It will provide elements likely to harmonize the care practices around the fixation of endotracheal tubes.

1. JUSTIFICATION OF THE STUDY

1.1. POSITIONING OF THE RESEARCH

Nearly 100,000 patients per year in France hospitalized in an intensive care unit for more than 48 hours (extrapolation from data from the REA-RAISIN surveillance network) require invasive respiratory assistance with the placement of an endotracheal tube, most often introduced via the oro-tracheal route.

Patients hospitalized in intensive care units are at risk of developing pressure ulcers, and prevention is an aspect of daily nursing care (6). With the development of new medical devices (MD), the prevention of medical device related pressure injuries (MDPI) has become a new focus for improving the management of intensive care patients.

The incidence of pressure injuries related to these systems varies in the literature from 11.9% (1) to 40% (2). Medical device-related pressure ulcer is defined as an area of localized injury to the skin or underlying tissue as a result of sustained pressure from devices designed and implemented for diagnostic or therapeutic purposes. The resulting tissue lesion has the shape of the medical device and has a propensity to progress rapidly due to minimal fatty tissue at the various sites of ulceration (1,2). The prevention of MD-related injuries is more complex and difficult than that related to pressure ulcers. This is because, in most situations, the maintenance of these medical devices is critical to survival (3).

The most common medical device injuries identified by ICU nurses were those related to endotracheal tubes (2,4,5).

The endotracheal tube is fixed to the face to ensure that it is maintained in an optimal position for effective ventilation. The correct positioning of the endotracheal tube is first checked by chest X-ray and then verified daily by the nurse using graduated markers. The fixation systems are numerous and differ according to the resuscitation services considered.

However, this fixation of the endotracheal tube to the face has the counterpart of generating an excessive pressure on the underlying skin, source of mucocutaneous lesions of the peri-oral region (8). Beyond the pain experienced, the resulting lesions can be unsightly, sometimes resulting in scarring.

This can have a psychological impact on the patients, with an alteration of the self-image causing anxiety, that can go as far as the social isolation of the patients. This can also give the family a negative image of the care given to the patient and goes against the notion of "taking care" which is at the heart of the nurse's role.

There are several types of lesions encountered in the peri-oral area: skin lesions, mucosal lesions and shearing/cuts. The pressure associated with the presence of ETT fixation systems is exerted over a prolonged period of time (on average 7 to 10 days for patients ventilated more than 48 hours). In addition, patients are frequently unable to move the tube in case of discomfort or to express their pain or discomfort (initial coma, deep sedation, etc.). There are risk factors for the development of pressure ulcers in severe resuscitation patients such as: sedation, decreased tissue perfusion, malnutrition, patients on vasopressors (6). Approximately 36% of ICU patients develop this type of peri-oral lesions during their hospitalization according to the prospective observational study conducted in 2011 under the aegis of the Société de Réanimation de Langue Française (7). Moreover, half of the lesions observed in this study appear between D1 and D5 of the hospitalization in intensive care unit.

The description of these lesions is done by the nurse who currently uses the NPUAP pressure ulcer scale to follow the evolution of the lesions. However, this scale cannot be used to follow the evolution of mucosal lesions. Indeed, these lesions are of a different nature, they do not have the same histological characteristics and the healing process differs (8). The "ROMPIS" scale was recently developed to evaluate mucosal lesions according to a classification allowing a better management of these specific lesions according to their stage (9).

Injuries related to these fixation systems develop for several reasons. First, the intubation tube is usually made of a rigid material that can cause friction or pressure on the underlying soft tissue. The adhesive tapes used to secure these devices can also irritate sensitive skin, and the cord can cause shearing or cutting, especially in the presence of edema, which is often the case in the ICU.

Several fixation systems co-exist: simple fixation methods not specifically developed as endotracheal tube fixation systems (plasters, adhesive elastic bands, etc.), or developed exclusively for this use (cord, cord+sheath, foam), or sophisticated systems developed by the pharmaceutical industry (such as AnchorFast, Marpac 320, Stabilock.....). Their purpose is to minimize the movement of the tube, to be able to be installed quickly and to create as little skin and mucous membrane damage as possible for the patient (10).

The impact of sophisticated devices developed by the pharmaceutical industry on the development of peri-oral lesions varies according to the studies. They are sometimes associated with an increase in peri-oral lesions (before-and-after study, published in 2018, on a group of 1100 patients evaluating a simple cord fixation versus the "AnchorFast ™") (11) or sometimes associated with a decrease in peri-oral lesions (single- center prospective study published in May 2019 comparing the "AnchorFast ™" system and adhesive strips) (12). Nevertheless, fixation systems developed by the pharmaceutical industry exert more pressure on the facial skin as suggested by a mannequin study comparing 10 sophisticated industrial systems and 6 "simple" systems (10).

Sophisticated systems developed by the pharmaceutical industry are rarely used in France. The prospective observational study carried out in 2011 under the aegis of the Société de Réanimation de Langue Française (7) also showed the heterogeneity of practices with the use of different fixation systems. The study indicated the predominant use of the fixation cord more or less associated with a protective sheath before that of an elastic adhesive band. At the beginning of 2019, we carried out a review based on a questionnaire for which 29 intensive care units reported their practice. Thus, among the 6 different fixation systems used, 2 stand out: the elastic adhesive tape (34.5%) and the cord associated with a protective sheath (24%).

Therefore, simple fixation systems are more often used in French ICUs. The divergent results concerning the cutaneous-mucosal impact of sophisticated fixation systems partly explain their low use in French intensive care units. Their high cost compared to simple systems is certainly also an obstacle to their use.

There is currently no formal recommendation for the use of a particular endotracheal tube fixation system. This encourages the heterogeneity of fixation practices in French ICUs.

The FIXATUB study is part of this context. Its objective is to evaluate the impact of the two fixation systems most commonly used in intensive care units in France (adhesive elastic band versus fixation cord with PVC sheath) on the incidence of peri-oral skin lesions. It will be the first multicenter randomized study on this subject. It will allow to bring elements of answer likely to modify and to homogenize the care practices and to make more rational the management of the intubated patients in intensive care unit on this aspect. The cluster study will allow an evaluation in real conditions of implementation of the two fixation systems evaluated.

The bibliographic references are listed at the end of the protocol.

1.2. BENEFITS AND RISKS FOR THE PERSONS FOR RESEARCH PURPOSES

1.2.1. Benefits

1.2.1.1. Individual benefit

The maintenance of the ETT during invasive respiratory support can be a source of peri-oral lesions. The aim of the research is to find a reliable fixation that causes the least peri-oral lesions while allowing an efficient maintenance of an optimal position of the ETT. These lesions cause physical pain that is increased during patient care.

Sometimes these unsightly lesions can also have a psychological impact on patients, with an alteration of self-image causing anxiety, anxiety that can go as far as the social isolation of patients. This can give the family a negative image of the care provided to the patient. Following this study, we hope to be able to improve this aspect of the management of critically ill patients.
The management between the centers will be standardized and the team will be sensitized to the management of pressure sores.

The person who participate to the clinical research will benefit from an increased monitoring of the presence of pressure sores with monitoring of the peri-oral cutaneous-mucosal state as of his entry in the intensive care unit with daily recordings and thus from a possibly earlier management with potentially less complications.

1.2.1.2. Collective benefit

The study will allow an optimization of the management of critically ill patients under invasive respiratory assistance.
This will potentially allow manufacturers to improve the systems currently on the market.

1.2.2. Risks

1.2.2.1. Individual risk

Both fixation systems are used on a daily basis in several intensive care units in France.
Prior to each period, teams will be trained on the new fixation system (when it differs from the usual system used) by the coordinator through oral presentations and films. A period of adjustment is planned before recruitment start.
The gestures will therefore have become routine and should not entail any additional risks.

1.2.2.2. Collective risk

The disposal of the waste is carried out according to the usual procedures for both groups, without additional risk or cost.

1.2.3. Benefit/risk balance

The research manager qualifies the research as **interventional research with minimal risks and constraints**, since :

- All procedures are performed in a routine manner and defined in the decree of April 12, 2018 set by the ministry.

Indeed:

- The fastening systems proposed in the study are both systems most commonly used in France based on our survey conducted in January 2019 in 29 French ICUs They are CE marked and will be used in accordance with their usual use in patients hospitalized in intensive care units
- The change of fixation is performed daily by a qualified nurse in the ICUs in France.

The research does not focus on techniques or strategies that are neither innovative nor obsolete.

All patient management will be identical to usual practice. In particular, the date of discharge will be decided by the doctor in charge of the patient, independently of the study, but will be reported in the patient's file and the research CRF.

Consequently, the particular modalities of implementation in the research represent negligible constraints for the person who participate to the research. (Article R 1121-3 of the public health code (CSP), decree n° 2006-477 of 26 April 2006).

The sponsor will submit the study protocole to the Comité de Protection des Personnes Ouest IV-Nantes (Ethics Committee), before any implementation of the research, for a favorable opinion and confirmation of the qualification of the research,in accordance with article L 1121-1 of the Public Health Code (CSP) as they result from the laws n° 2004-806 of August 9, 2004 and n°2006-450 of April 18, 2006 relating to the public health policy.

2. OBJECTIVES AND EVALUATION CRITERIA

2.1. *OBJECTIVE AND MAIN EVALUATION CRITERION*

2.1.1. Main objective

To demonstrate that the elastic adhesive tape fixation strategy decreases the risk for developing a peri-oral lesion before the 10th day of maintaining the orally inserted ETT.

2.1.2. Primary endpoint

Appearance of at least one peri-oral lesion during the first ten days of maintaining the orally inserted endotracheal tube.
The peri-oral lesion will be validated on photographs by an independent review committee. The photos will be taken daily, taking the precaution that the fixation system is not visible (maintaining the blindness for the adjudication committee) from D0 to D10.

2.2. *SECONDARY OBJECTIVES AND EVALUATION CRITERIA*

2.2.1. Secondary Objective(s)

- Verify the effectiveness of the device in maintaining the endotracheal tube in terms of self-extubation from intubation until extubation or D28.
- Verify the effectiveness of the device in maintaining the endotracheal tube in terms of repositioning the tube from intubation until extubation or D28.
- Compare the time to the first peri-oral lesion from intubation to extubation or D28.
- Compare the intensity of peri-oral skin lesions assessed by nurses from intubation till extubation or D28.
- Compare the intensity of mucosal peri-oral lesions assessed by nurses from intubation till extubation or D28.

- Assessing the incidence of peri-oral lesions
- Evaluate the impact of the intervention on the maximum number of lesions per day.

2.2.2. Secondary endpoint(s)

- Number of self-extubations
- Number of significant repositioning of the endotracheal tube performed following a movement of more than 2 cm from the initially set marker.
- Time to the first peri-oral lesion from the time the patient is intubated until extubation.
- Assessment of the grade of cutaneous peri-oral lesions by the NPUAP scale.
  If several lesions co-exist, the lesion with the most severe grade will be retained.
- Evaluation of the intensity of the mucosal peri-oral lesions by the ROMPIS scale. If several lesions co-exist, the lesion with the most severe grade will be retained.
- Number of patients with at least one peri-oral lesion per 1000 ventilation days
- Maximum number of lesions per day

3. STUDY POPULATION

3.1. *DESCRIPTION OF THE POPULATION*

The inclusion target is 768 patients in 16 investigating centers, i.e. 24 patients per center per inclusion period (see paragraph 6.2.1 Description of planned statistical methods).

Included patients will be patient admitted in ICU, requiring artificial ventilation with an orotracheal ETT for an estimated duration of more than 48 hours, as well as the use of vasopressors, and who will meet the inclusion and non-inclusion criteria described below.

Indeed, the fact that the patient is on vasopressors is an additional risk factor for pressure ulcers. The addition of this criterion makes the population more homogeneous. The results of this study could be extended to patients not on vasopressors.

In terms of potential recruitment, in the DEMETER project (study promoted by the CHD Vendée, NCT02515617), 10 of the 16 centers involved in FIXATUB study recruited 927 patients in 20 months (i.e. 741 in 16 months). The patients included in the DEMETER project met almost the same inclusion criteria as in FIXATUB. Thus, the recruitment of 3 intubated patients per month seems easily achievable in view of the population admitted to the ICU.

3.2. *INCLUSION CRITERIA*

- Patient hospitalized in intensive care
- Age over 18 years
- Intubation for an estimated duration > 48 hours
- Patient treated with vasopressors
- Patient or family member who has received the information and does not object to participation in the study or patient included under the emergency procedure in the absence of contactable family members

3.3. *CRITERIA FOR NON-INCLUSION*

- Pre-existing facial lesions on arrival in the intensive care unit on the path of the endotracheal tube fixation
- Admitted intubated following transfer from another intensive care unit
- Nasotracheal intubation
- Patient in isolation for suspected COVID or clinically proven COVID
- Patient admitted with tracheostomy
- Pregnant, nursing and parturient women
- Lack of social security affiliation
- Moribund (High probability of death within 48 hours of inclusion)
- Incapacitated adult (under guardianship, curatorship)
- Patient deprived of liberty by court order

4. DESIGN AND CONDUCT OF THE STUDY

4.1. GENERAL METHODOLOGY OF THE RESEARCH

- Interventional study with minimal risks and constraints
- National multicenter study
- Controlled study
- Cluster cross-over randomized trial
- Open study

The choice to conduct a cluster trial is based on the following two elements:

- Endotracheal intubation of patients is most often performed in an emergency context which does not allow time for a possible randomization and use of the ETT fixation system allocated by individual randomization.
- The studied intervention goes beyond the simple use of a specific fixation system: it affects practices. Some centers are not currently using the fixation systems under study and will need to be trained in their use, which justifies a collective randomization unit for a good implementation of this new care practice. This also reduces the risk of error or poor implementation of the system due to the coexistence of several systems in the same department. Group randomization is classically used when we are interested in interventions aiming to impact practices.

The choice of the crossover is justified by :

- The power gain induced by the crossover, compared to a parallel group trial
- The gain in comparability of the groups that can be expected, due to the limited number of clusters (i.e. resuscitation centers) participating.
- The absence of a possible residual effect of the study intervention. Indeed, the endotracheal tube fixation systems will be different in each of both periods.
  At the end of the period, the previous fixation systems system will be removed from the arsenal of equipment available in the department.

The photos taken daily for each included patient will then be reviewed by an independent committee (2 nurses, 1 or 2 dermatologists) who will assess the presence or absence of a peri-oral lesion.

4.2. DIAGRAM OF THE STUDY

- Duration of inclusion: 2 periods of 12 months with a 4-month break
- Duration of study participation for a patient: 28 days after inclusion
- Number of patients to be included: 768 patients
- Total duration of the study: 29 months

Each patient will be followed up for up to 28 days after inclusion with vital status at discharge.

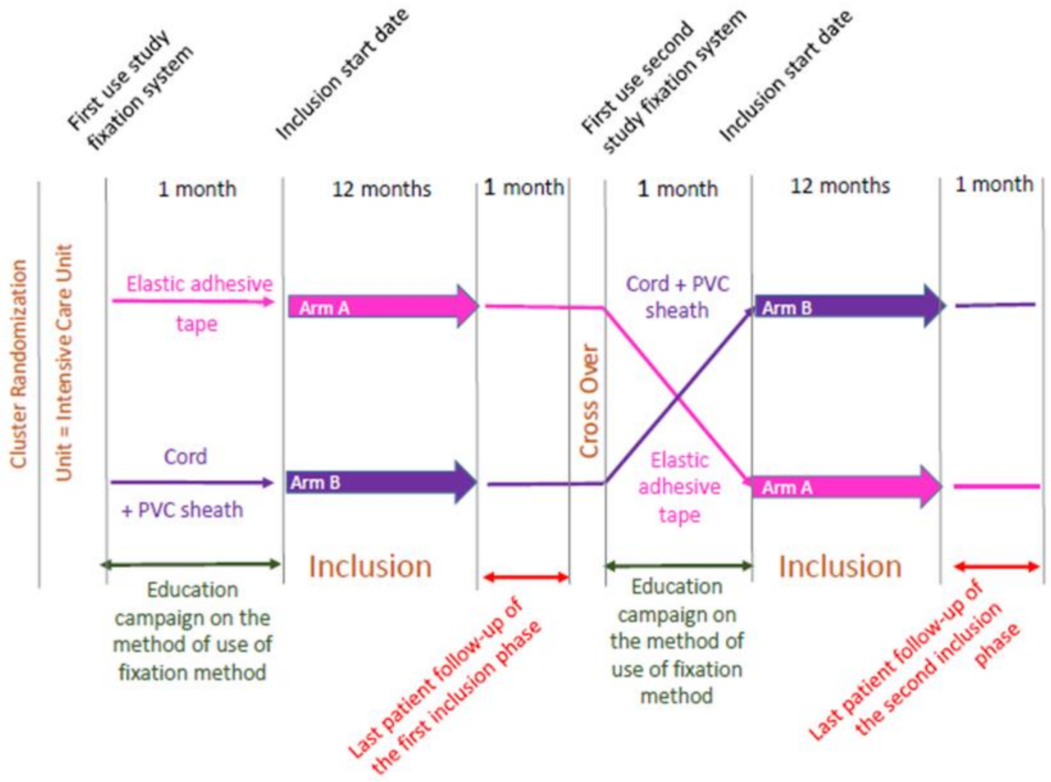

4.3. STUDY SCHEDULE

The study will include two 12-month inclusion periods with a 4-month interruption period. Each of the recruitment periods will cover one year, which will allow us to avoid the effect of seasonality.

Each department will be assigned an A or B arm by randomization before the start of the study (see diagram above). Before the fixation systems are sent to the centers, information will be given to all the medical and paramedical staff in each investigating center on the use of the two fixation methods that will be used during the study. This information will be given through a slide show and in the form of triptics which will be available in each department. Explanatory videos of the methods of use of both fixation systems will also be made for each investigating center. This information will be delivered over several sessions in order to reach the whole team.
One month before the start of enrollment, the investigating centers that will start with arm A will be equipped with elastic adhesive tape fixation so that the fixation procedure is integrated into the management of patients in these centers and the paramedical teams take ownership of this strategy. Centers starting with arm B will be equipped with cord fixation in a sheath.

Start of inclusions: first phase of inclusion with 8 centers in arm A and 8 centers in arm B.
Thus, in arm A, the patients who will be intubated will have elastic adhesive bands and in arm B the patients who will be intubated will have fixations with a cord in a pvc sheath.
Follow-up of patients in the first period will end no more than 28 days after the last inclusion.

One month before the start of the second period, the investigating centers that will end the period in arm B will be equipped with elastic adhesive bands. As for the centers that will end the period in arm A, they will be equipped from this date with the fixations by a cord in a PVC sheath. The new fixations will be sent to the centers beforehand in order to be ready to start the new phase of the information campaign with the investigating centers' health care teams.

In addition, a new education campaign on the use of both fixation system will be launched in the same manner as the previous year.
Start of the second inclusion period: second inclusion phase with 8 centers in arm A and 8 centers in arm B.

The inclusion periods will be separated by a 4-month interval. This intermediate period will allow for the completion of the one-month follow-up of patients from the first period without changing the fixation system used in the center. For the following 2 months, centers will remain with the fixation systems from the first period or may use their usual system if they wish. The second fixation system of the project will then be compulsorily used in the departments during the month preceding the beginning of the 2nd inclusion period.

Inclusion

This trial relates to care practice. In order to meet the main objective, it requires homogeneity of intubation fixation practices within a given department during each of both inclusion periods, regardless of the status of the patient in the study (included or not).

Thus, in each ICU participating in the project, whatever the inclusion arm considered (A or B), all patients requiring invasive mechanical ventilation (MV) from their admission to the intensive care unit (if the respiratory assistance was set up before admission to the intensive care unit) or from the moment the respiratory assistance was set up (if it was set up in the intensive care unit), will benefit from the fixation method according to the inclusion arm (A or B) described above, whether they are included in the study or not. D0 is defined as the first day of the study fixation system of the ETT (cord + sheath or elastic adhesive bands).

Information and consent to participate in the study will be sought from patients requiring MV (or their relatives), who meet all the inclusion criteria and have no non-inclusion criteria (see chapter 3.3).
In the absence of the physical presence of the trusted support person or a relative, an emergency procedure will be available to include the patient. In all cases, the relative and then the patient will be informed as soon as possible by the investigator who will deliver the information note and the collection of their oral consent will be carried out secondarily by the investigator.

After having ensured the consent of the patient or his relatives or having followed the emergency inclusion procedure, the investigator can then include the patient before the D1 photograph. Moreover, if the information was initially given to the relatives, the consent will also be sought from the patient as soon as his level of vigilance allows it.

One of the criteria for non-inclusion of the patient is the presence of pre-existing facial lesions on arrival in the ICU in the path of the endotracheal tube fixation.

When the patient is admitted to the ICU already intubated, the tube is already fixed and this fixation is generally changed in order to put in place the fixation system used in the department. Within the framework of the protocol, we will ask that it be systematically changed to install the fixation system under study. The non-inclusion criterion can therefore be assessed at this point.
Assessment of the primary endpoint requires a photograph of the patient's mouth upon admission. The patient is often unconscious and/or unable to give consent. Relatives are not always present to be informed of the study and to give their consent. Waiting for the consent of the relatives requires removing the fixation system again after having obtained their consent in order to take the picture. This may result in additional skin damage. In addition, it is necessary to take the photo of each patient at the same time (admission) so that each patient has an initial photo with a comparable temporality. If relatives come 24 hours after admission, a lesion could possibly appear when there was none at admission.
We therefore propose to take a photograph of all intubated patients on admission and to delete this photograph from the device if the patient is ultimately not included in the FIXATUB study.

A follow-up table of patients likely to participate in the study will be kept up to date, noting the reasons for non-inclusion and objections to participation. This will be sent to the coordinating investigating center at the beginning of each month in a format that respects patients' anonymity.

Follow-up

During the two recruitment periods, a logbook will be given to the paramedical staff and will include the follow-up of each change of fixation made during their stay in the ICU.
The notebook will also record the presence of vasopressors, prone positioning, extubation or re-intubation, or the presence of sedation, which are risk factors for injury in resuscitation patients.
The nurse in charge of the patient will have to change the fixation system at least once a day and if necessary during the day in case of soiling or examinations.
Duoderm-type dressings used to prevent pressure sores under the endotracheal tube will not be accepted, since this is the subject of the study. However, it can be applied as a curative measure if a lesion appears.
A verification of the correct positioning of the endotracheal tube thanks to the graduated marker will be systematically carried out once a day. If a repositioning is necessary the nurse will note the number of centimeters (either at the bottom or at the top). The mobilization of the tube (change of side) will be performed according to the usual practices of the service.

The number of mobilizations, interventions (e.g., retying the knot) on the fixation system and the number of fixation system changes will be recorded daily.

The presence or absence of lesions will be assessed daily by the nurse in charge of the patient. She will also assess the type of lesion: cutaneous or mucous and depending on the type will use the appropriate scale to assess the grade: NPUAP scale (cutaneous) or / and the ROMPIS scale (mucous).

Two photos (one with the lower lip visible and one with the upper lip visible) will be taken every day by the nurses from the day of the first fixation change = D0 until D10. In no case will the whole face of the patient be taken. The patient will not be identifiable. The photos will be taken without the fixation visible to allow the blind to be kept for the committee that will evaluate the photos. The photos will be identified only by the patient's number and the date.

Each expert will have to record the presence or not of a peri-oral lesion between D0 and D10 of maintenance of respiratory assistance via an endotracheal tube. .
For the primary endpoint, we selected a daily photo assessment until D10. The choice of this time frame was based on:

- the time of appearance of peri-oral lesions (more than 50% of them appear during the first 5 days) (7)

- the average duration of ventilation for patients who require invasive respiratory assistance for more than 48 hours
- risk factors for pressure ulcers which are essentially present at the beginning of the management of patients in shock (oedema, sedation, decrease in tissue perfusion, undernutrition, vasopressor treatments)

The follow-up by the nurse's notebook will stop at the final extubation or at the discharge from the ICU if the patient is discharged intubated from the ICU before D28 or at most D28.

SCHEDULE OF THE STUDY

Actions	D0	D1	D2	D3	D 4	D5	D6	D7	D8	D9	D10	D22 to D27	D28
Inclusion / non-inclusion criteria	X												
Participation agreement	X												
Biological assessment for SOFA	X												
**Assessment of peri-oral lesions:**													
Paramedical examination (peri-oral lesion survey)	X	X	X	X	X	X	X	X	X	X	X	X	X
Photographs (during the change of the fixation system)	X	X	X	X	X	X	X	X	X	X	X		
Collection of endotracheal tube mobilizations	X	X	X	X	X	X	X	X	X	X	X	X	X
Collection of the repositioning of the endotracheal tube	X	X	X	X	X	X	X	X	X	X	X	X	X
Collection of re-intubations	X	X	X	X	X	X	X	X	X	X	X	X	X
Collection of adverse events related to the investigation procedure and incidents related to the MD	X	X	X	X	X	X	X	X	X	X	X	X	X

4.4. *DESCRIPTION AND JUSTIFICATION OF THE INTERVENTION STUDIED*

The FIXATUB study is a cluster and crossover randomization study. Patients will be included over 2 periods of 12 months. The timing of both periods (period with the use of the elastic adhesive tape and period with the use of the cord associated with a protective sheath) will be randomly assigned for each center. The sponsor will provide the fixation systems for each period.

There are 3 cord + sheath type fastening systems currently available:
Teleflex® ETT fixation straps, Int'air® medical straps and CairLGL® fixation laces. All 3 are made of cotton tape and a flexible PVC protective sheath and are all 3 classified as MD class 1 non-sterile. No data is available to evaluate the superiority of one of these sheaths.
Each center provided us with their annual consumption of ETT fixation systems in order to obtain a precise evaluation of the quantity of devices required for the project. The quantity of sheaths required being important and the sheath being more expensive than the adhesive elastic band, we will proceed to an "adapted" tender procedure for these 3 suppliers and the devices being similar, the least expensive offer will be chosen.

As for the elastic adhesive tape, we will carry out a competition between the various suppliers and will opt for the least expensive.

Description of fastening techniques

Arm A: "elastic adhesive tape

Fixation by elastic adhesive tape (Tensoplast type adhesive tape):
The elastic band must be cut in 4 and then in 2 in the width to obtain 8 bands. The length obtained will be 62-63 cm and about 1.5 cm wide.
The tape will be attached to the patient's face (opposite side of the endotracheal tube) and then two wraps around the endotracheal tube. The rest of the tape will be attached to the other side of the face (endotracheal tube side).
The plasticized strip will be kept on the adhesive strip the time to pass on the nape of the neck in order not to stick to the hair.

Finally, the end of the adhesive tape will be replaced on the part already attached to the patient.

Arm B: "cord + sheath

1) Pull the loop (in the middle of the fastener) to loosen it

2) Position the loop around the ETT tube from below.

3) Pass the PVC part of the fastener through the loop.

4) Pull the ends of the cord out of the sheath to tighten the loop around the ETT tube.

5) Tie a knot with both ends of the cord so that the fixation systems is secure around the patient's head

Each fixing systems will be changed daily and after soiling or examinations if necessary.

4.5. DESCRIPTION OF ASSESSMENT AND OF DATA COLLECTED

4.5.1. Detailed description of evaluation parameters

NPUAP scale:

This scale allows the evaluation of pressure sores according to 4 stages graduated from 1 to 4. Stage 1 being the least severe. The National Pressure Ulcer Advisory Panel's (www.npuap.org) Stages scale translated from English by ANAES will be used.

stade		description
I	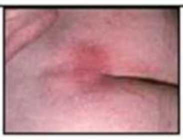	Le premier stade est une altération observable d'une peau intacte, liée à la pression et se manifestant par une modification d'une ou de plusieurs des caractéristiques suivantes en comparaison avec la zone corporelle adjacente ou controlatérale : température de la peau (chaleur ou froideur), consistance du tissu (ferme ou molle) et/ou sensibilité (douleur, démangeaisons). Chez les personnes à la peau claire, l'escarre apparaît comme une rougeur persistante localisée, alors que chez les personnes à la peau pigmentée, l'escarre peut être d'une teinte rouge, bleue ou violacée persistante.
II	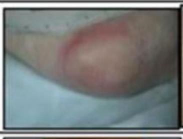 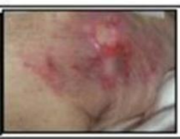	Perte d'une partie de l'épaisseur de la peau; cette perte touche l'épiderme, le derme ou les deux. L'escarre est superficielle et se présente cliniquement comme une abrasion, une phlyctène ou une ulcération peu profonde.
III	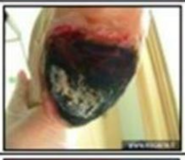	Perte de toute l'épaisseur de la peau avec altération ou nécrose du tissu sous-cutané ; celle-ci peut s'étendre jusqu'au fascia, mais pas au-delà. L'escarre se présente cliniquement comme une ulcération profonde avec ou sans envahissement des tissus environnants.
IV	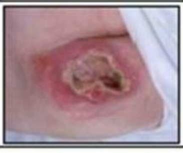	Perte de toute l'épaisseur de la peau avec destruction importante des tissus, ou atteinte des muscles, des os, ou des structures de soutien (par exemple des tendons, des articulations). Un envahissement et des fistules peuvent être associés au stade IV de l'escarre

ROMPIS scale:

To assess the intensity of mucosal injury, The Reaper Oral Mucosa Pressure Injury Scale (ROMPIS) will be used. First published in 2017 (9), the ROMPIS scale is the only scale to assess mucosal injury in this setting.

Stage	Descriptor
1	Redness and demarcation of the lip and buccal mucosa, with no visible destruction or loss of epithelial tissue, ulceration or blisters. Non-blanchable erythema on the corners of the mouth.
2	Destruction and differentiation of buccal mucosa, as manifested by blisters, soft coagulum, or clotting on mucosal tissue; superficial loss of non-keratinised epithelial tissue; or damage to epidermal and dermal layers of the corners of the mouth, without evidence of damage to underlying fascia.
3	Loss of mucosa and sub-mucosal tissue as evidenced by damage to/exposure of the fascia and underlying muscle in the lips or corners of the mouth.

Adjudication Committee:

Several dermatologists and 2 nurses independent of the study will compose the adjudication committee. Each file (including all the photos of the same patient) will be randomly transmitted to each member of the adjudication committee. The members will rule on the presence or

absence of a lesion. If there is a discrepancy between the opinions of the committee members (if at least one of the experts disagrees with the other two), the committee will meet to discuss the matter and decide together whether or not the lesion is present.

4.6. *IDENTIFICATION OF ALL SOURCE DATA NOT INCLUDED IN THE MEDICAL RECORD*

Daily :

-Peri-oral lesions according to the NPUAP and ROMPIS scales

-Number of endotracheal tube mobilizations

-Presence or not of a protection of the ETT tube by specific system (guedel or mouthpiece)

Data related to photo adjudication: presence or absence of peri-oral lesions

4.7. *RULES FOR TERMINATING A PERSON'S PARTICIPATION*

4.7.1. Criteria for premature termination of an individual's participation in research

An individual's participation may be terminated prematurely for the following reasons:

- Withdrawal of consent by the patient

Subjects will be able to withdraw their consent and ask to leave the study at any time for any reason.

- Transfer to another intensive care unit

4.7.2. Monitoring procedures and data collection schedule

In case of withdrawal of consent by the patient, and without prejudice to the patient's right; the data already collected will be analyzed unless the patient objects.

No other examinations specifically provided for in the protocol will be performed and no data will be used in accordance with the patient's wishes after withdrawal of consent.

The discharge of a patient from the study will not change the patient's usual management of his or her pathology.

4.7.3. Discontinuation of part or all of the research at the sponsor's discretion (excluding biostatistical considerations)

Part or the whole study may be stopped permanently or temporarily by decision of the ANSM, the CPP, or the Study Sponsor.

In all cases:
- A written confirmation will be sent to the coordinating investigator of the study (specifying the reasons for premature termination) as well as to the principal investigator of each center if applicable.

5. SAFETY ASSESSMENT

This protocol is subject to an update of the Safety paragraph in accordance with European Regulation n°2017/745 of the European Parliament and of the Council of 5 April 2017 concerning medical devices (MDR) and the ANSM's Notice to Sponsors (*Clinical Investigations of MD, Part IV, Vigilance).* The vigilance of this study therefore follows the provisions **of Articles 87 to 90 of the MDR (Material Vigilance)** for any incident involving the MD and **Article 80 only for adverse events related to the investigation procedure.**

5.1. DEFINITIONS

According to the MDR, Medical Device Coordination Group (MDCG) 2020-10/1, ANSM Notice to Sponsors (*Clinical Investigations of MDs, Part IV, Vigilance)* and NF EN ISO 14155 Clinical Investigation of MDs for Human Subjects-Good Clinical Practice:

**Adverse Events (AEs)**<br>MDR, Article 2 (57)	Any harmful event, unintended illness or injury, or untoward clinical sign, including an abnormal laboratory result, in participants, users, or others in a clinical investigation, whether or not related to the device under clinical investigation.<br><br>Notes (NF EN ISO 14155): This definition includes events related to the medical device under investigation or the comparator.<br>This definition includes the events related to the procedures involved.<br>For users or other persons, the definition refers only to events related to the use of the device under investigation or comparators.
**Serious adverse events (SAE)**<br>MDR, Article 2 (58)	Any adverse event that resulted in:<br><br>a) death;<br><br>b) a serious deterioration of the participant's health condition, which causes :<br><br>- a life-threatening illness or injury;<br><br>- a permanent impairment of an anatomical structure or function;<br><br>- hospitalization or prolongation of the patient's hospitalization ;<br><br>- medical or surgical intervention to prevent illness or injury that endangers

	the patient's life or any permanent impairment of an anatomical structure or function;<br>- of a chronic disease;<br>(c) fetal suffering, fetal death, congenital physical or mental impairment or congenital malformation.<br>Note (Standard NF EN ISO 14155): A procedure required by the clinical investigation plan, without serious deterioration of health, is not considered as an event serious adverse event.
**New fact**	Any new data that may lead to a reassessment of the benefit/risk ratio of the research or the product under investigation, to changes in the use of the product, in the conduct of the research, or in the documentation of the research, or to the suspension or discontinuation or modification of the protocol of the research or similar research. For trials involving the first administration or use of a health product in individuals who do not have a medical condition: any effect<br>serious adverse event.
**Incident**<br>MDR, section 2 (64)	Any malfunction or alteration in the characteristics or performance of a device made available on the market, including an error in use due to ergonomic characteristics, as well as any defect in the information provided by the manufacturer and any undesirable side effect.
**Serious incident**<br>MDR, section 2 (65)	Any incident that directly or indirectly resulted or may have resulted or may have resulted in:<br>a) death of a patient, user or any other person;<br>b) a serious deterioration, temporary or permanent, in the state of health of a patient, user or any other person;<br>c) a serious threat to public health.
**Investigation procedure** (preceding or concomitant with the SAE)<br>Notice to Sponsors from ANSM<br>the SAE)	All activities related to the use of the MD that are the subject of the study, imposed by the study protocol implemented before the occurrence of the adverse event or concomitantly and for which a causal relationship with this event is suspected. This notion includes, but is not limited to, additional invasive or cumbersome procedures in relation to the routine use of the device.<br>For example: the surgical technique related to the placement of an implantable MD subject to a study, the use of imaging to monitor the placement of the MD studied.
**Serious threat to public health**	An event that may result in an imminent risk of death, serious deterioration of health or illness<br>that may require prompt corrective action, and

MDR article 2 (66)	likely to result in significant human morbidity or mortality or which is unusual or unexpected at the time and place of occurrence.
**Special situations**<br>- Abuse	Intentional, persistent or sporadic excessive use of drugs or products mentioned in article R. 5121-150 of the Public Health Code (CSP), accompanied by harmful physical or psychological reactions.
-Medication error	Unintentional error by a healthcare professional, a patient or a third party, as the case may be, occurring during the care process involving a medicinal product or a healthcare product mentioned in article R. 5121-150 of the CSP, in particular during prescription, dispensing or administration.
- Misuse	Intentional and inappropriate use of a drug or product that is not in accordance with the marketing authorization or registration and good practice recommendations.
- Overdose	Administration of an amount of drug or product, per dose or cumulatively, that is greater than the maximum dose recommended by the summary of product characteristics referred to in Article R. 5121-1, which is the subject of a clinical opinion.
**User**<br>MDR, section 2 (37)	Any health care professional or lay person who uses a device.

5.2. *SPECIFICITIES OF THE PROTOCOL*

5.2.1. Investigation procedure

The investigation procedure in this protocol corresponds to the placement, maintenance, daily change and removal of the attachment system (elastic adhesive band or cord + sheath) of the endotracheal tube.

5.2.2. Adverse events / Expected incidents

- List of Incidents/Expected Events related to the investigational medical device/comparator

- *The medical device under study (elastic adhesive tape):*

The MD is used in its indication: fixation.

(There is no detailed AE in the technical data sheet except for one contraindication: subjects sensitized to synthetic products).

Expected Incidents / AE:

- Intolerance to synthetic products
- Skin lesions and mucosal lesions in the path of fixation. (*13% of patients according to reference 7)*
- Loss of adhesiveness of the tape.

- The medical device comparator (cord + sheath):

The comparator is used in its indication: fixation.
There are no AE detailed in the technical data sheet.

Expected Incidents / AE:

- Skin and mucosal lesions in the path of fixation (*43% of patients in reference 7)*
- Shearing, potential cut due to cord.

- List of expected AEs related to investigative procedures:

- Medical device implementation:

- Unscheduled extubations: self-extubations and accidental extubations during care *(7 to 16% of patients depending on reference 17)*
- Tracheal complications including laryngeal dyspnea
- Ventilator-associated pneumonia.

- List of expected AEs related to other additional procedures:

- Auxiliary Drugs/MDs:

- *Anti-adhesive (elastic adhesive tape group) to remove residual glue.*
- *Duoderm ® or other dressings used in curative treatment in case of lesions.*
- *Cavilon ™ type skin protection film.*

As these treatments are prescribed within the scope of their indications, any AEs for them are described in the respective summary of product characteristics (SPC)s, which serve as a reference.

- *To the procedures performed, methods/techniques used for the purpose of the research:*

NA

- List of AEs related to the studied disease (death due to the disease, relapse, ...) :

The patients included will be patients hospitalized in the intensive care unit, most often with multiple pathologies, who are intubated and put on invasive mechanical ventilation.

The potential complications of intensive care management of the study patients are identical to those expected in the non-study population, including, but not limited to, nosocomial infections, complications of intubation procedures, anesthesia drugs, consequences of prolonged bed rest, occurrence of shock with a risk of multi-visceral failure, and death.

These adverse events will be considered to be related to the disease being studied and not to the medical device being studied or to the investigative procedure.

5.2.3. Events of interest

Within the framework of the evaluation of both MD (cord + sheath and adhesive elastic band) the **events of interest** induced by these MDs will be collected.

These are:

- Self-extubations
- Laryngotracheal dyspnea
- Ventilator-associated pneumonia

5.2.4. Non-serious adverse events not subject to registration

Complications due to concomitant treatments, which are therefore not imposed by the protocol, cannot be considered as events related to the MD, the comparator or the investigation procedure and will therefore not be entered in the eCRF. They must be transmitted to the regional pharmacovigilance center (CRPV).

Complications related to routine procedures (diagnostic or therapeutic) implemented as part of the patient's usual management outside of the CI protocol cannot be considered as events related to the MD, comparator or investigative procedure and will therefore not be entered into the eCRF. They must also be transmitted to the regional pharmacovigilance center.

5.2.5. Serious Adverse Event not submitted to registration and notification to the sponsor

Some circumstances requiring hospitalization do not fall under the severity criterion "hospitalization/extension of patient's hospitalization" and should not be recorded in the eCRF and notified to the sponsor as SAE :

- Hospitalization for medical or surgical treatment scheduled before the beginning of the research or usually performed and concerning a previously known pathology not related to the research and not associated with a deterioration of the patient's condition (e.g.: change of catheter, probe, implantable chamber, extraction of wisdom teeth, etc.).

5.3. *ROLE OF THE INVESTIGATOR*

The investigator is responsible for the registration via an entry in the e-CRF:

- Any **serious and non-serious adverse events** that **may be related to the preceding investigation procedure**
- **Serious and non-serious incidents** (or risks of incidents) that may be related to the MD
- Events of interest (section 5.2 of the protocol)
- Special situations even in the absence of complications and severity criteria
- New Facts

For each adverse event recorded on the e-CRF, the investigator will make an independent assessment of severity (intensity), causality and seriousness.
The investigator will seek information about adverse events at each encounter with the patient. The investigator will seek, whenever possible, to provide the definitive medical diagnosis.
All adverse events, whether reported by the patient or noted by study personnel, will be noted in the patient's medical record.
Exception: Peri-oral skin and mucosal lesions related to the fixation system are not to be collected in the CRF adverse event table. They are already included in the CRF follow-up pages. Other lesions outside of this area should be entered in the CRF adverse events page.

5.3.1. Assessment of the intensity of adverse events

The Common Terminology Criteria for Adverse Events (CTCAE) version 5, Nov. 2017 will be used to assess the intensity of adverse events.
The table below will be used to assess the intensity of adverse events that are not specifically listed in the CTCAE.

Grade	Intensity
1	Mild; asymptomatic or mild symptoms; clinical observations or diagnostic only
2	Moderate; minimal, local or non-invasive intervention indicated; or limitation age-appropriate instrumental activities of daily living [a]
3	Severe or medically significant, but not immediately life-threatening; hospitalization or extension of hospitalization indicated; disabling; or limiting activities of daily living [b,c]
4	Life-threatening consequences or urgent intervention indicated [c]
5	Deaths related to an adverse event [c]

[a] Instrumental activities of daily living refer to preparing meals, shopping or buying clothes, using the telephone, managing money, etc.
[b] Examples of personal care activities of daily living include bathing, dressing and undressing, eating, using the toilet, and taking medications as performed by patients who are not bedridden.
[c] **Grade events** likely to be **assessed as "significant medical event", and grade 4 and 5 events should be reported as serious adverse events.**

5.3.2. Evaluation of the causality of adverse events

The investigator should use his or her knowledge of the patient, the circumstances surrounding the event, and an assessment of any other potential causes to determine whether the adverse event is considered to be related to the study MD or the investigative procedure.
The following guidelines should be considered:

- Temporal relationship between the onset of the event and the first use of the MD under study

- Course of the event, with particular attention to the effects of discontinuing the study MD or reintroducing the study MD (if applicable)
- Known mechanism of action at the MD
- Body site or organ that may or may not be affected by the MD or condition
- Known association of the event with the investigational MD or similar devices
- Known association of the event with the disease under study
- Presence of risk factors in the patient or use of concomitant medications known to increase the frequency of the event
- Presence of factors unrelated to the MD that are known to be associated with the occurrence of the event.

Causality of MDs according to MDCG 2020-10/1 page 9

Not related	Causality with the MD, comparator or procedures can be excluded
Possible	Causality with the use of the MD, comparator, or CI procedures is weak but cannot be completely ruled out. Other causes are also possible (underlying or concomitant disease/clinical condition/effect of another treatment). Cases where the relationship cannot be assessed or no information has been obtained should also be classified as possible
Probable	The causal link to the use of the MD or comparator, or the relationship to the CI procedures, appears relevant and/or the event cannot be reasonably explained by another cause
Some	The serious adverse event is related to the use of the MD, comparator, or trial procedures beyond a reasonable doubt.

The categories "possible", "probable" and "certain" correspond to a proven or reasonably foreseeable causal link.
In cases where no information is available to define the causal link, fill in
The default setting is "possible".

5.3.3. Evaluation and Reporting of Serious Adverse Events and Incidents

The regulations have instituted a different notification circuit depending on whether an event/incident is suspected to be related to the MD itself or to the investigation procedure.

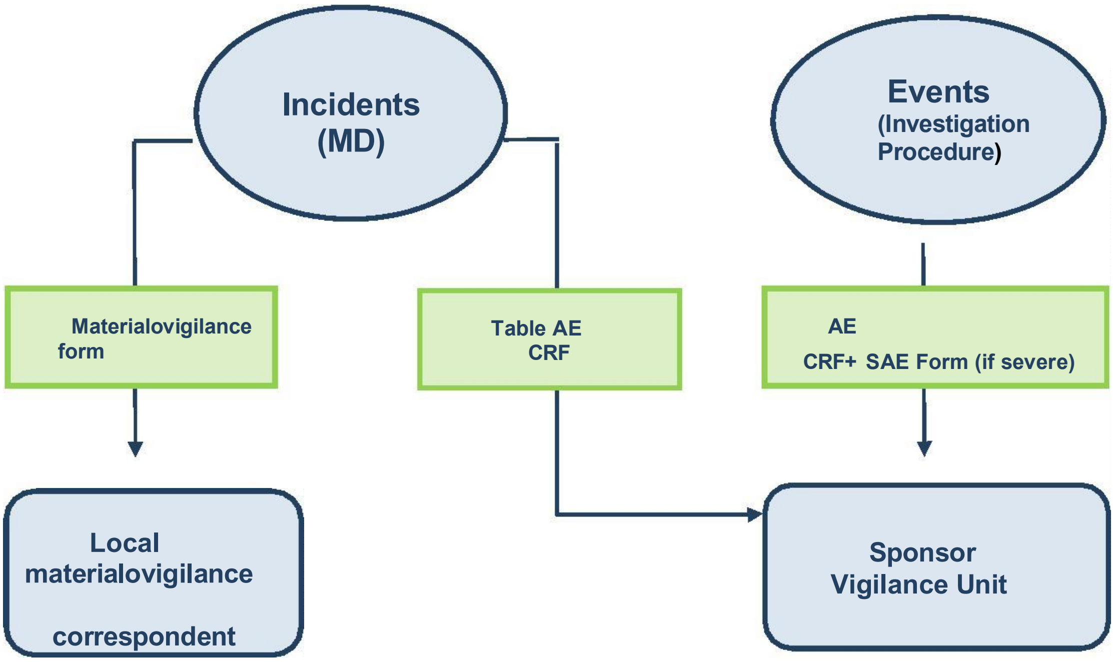

a. **Incidents (or risks of incidents) that may be related to the medical device (MD)**

They must be notified by the investigator to the **local materialovigilance correspondent of the institution in which the study is being carried out,** without delay and as soon as he becomes aware of them. **They follow the standard materialovigilance circuit of the institution, under the responsibility of the investigator (care circuit).**

The form dedicated to material safety is not to be sent to the sponsor, the mention of these AEs/incidents in the eCRF table being sufficient. The investigator should indicate in the eCRF that the transmission has been made to the local correspondent, via the checkbox in the table of AEs/Incidents.

The sponsor ensures that the required notifications have been made to the local Material Safety Correspondents of the investigating centers.

b. **Serious adverse events** in relation to **the investigation procedure that precedes it** according to the investigator

The investigator must report to the sponsor, without delay and at the latest **within 3 calendar days from the** day he/she becomes aware of them, all serious adverse events of the investigation procedure that preceded them that occurred in the study.

This initial notification should be followed by a detailed follow-up. The investigator must document the event as well as possible (thanks to copies of laboratory results, test reports and/or hospitalization reports giving information on the serious event, including relevant negative results, **without omitting to make these documents anonymous** and to write down: acronym of the research, number and initials of the patient, the medical diagnosis and establish **a causal link** between the serious adverse event and the medical device(s) and the action taken to implement it) **and, if possible, give the medical diagnosis.**
The investigator will complete and sign the paper SAE notification form and send the scanned form by email to [redacted]. He/she should specify in the subject line "NAME OF THE STUDY-Patient-Center".
The investigator follows any serious adverse event until complete resolution (disappearance of signs and symptoms), or consolidation (return to a medically acceptable state) even if the patient has stopped the research procedure, **and communicates to the sponsor any additional information** concerning this event by means of follow-up reports.

5.3.4. Procedures and deadlines for notifying the sponsor

The investigator notifies the sponsor of events as defined below:

TYPE OF EVENT	METHODS OF NOTIFICATION	NOTIFICATION DEADLINE
**Event of interest**	Collection in the e-CRF (AE table)	**Without delay** as soon as you are aware of it, and at the latest **within 3 calendar days**
**Non-serious adverse event** Suspected of being related to the clinical investigation procedure	Collection in the e-CRF (AE table)	As soon as you are aware of it
**Serious adverse event** Suspected of being related to the clinical investigation procedure	Collection in the e-CRF (AE table)<br><br>+ Initial SAE form<br>+ Follow up if necessary<br>By mail	**Without delay to the Sponsor,** as soon as you are aware of it, and at the latest within **3 calendar days**
**New Fact**	By email	**Without delay to the Sponsor,** as soon as you are aware of it, and at the latest within **3 calendar days**
**Incidents (MD)**	Collection in the e-CRF (AE table) + checkbox in the eCRF (AE table)<br>+<br>Materialovigilance declaration form	**Without delay to the local** materialovigilance **correspondent**, as soon as he is aware of
**Special situation**	SIP form to be completed + Follow up if necessary<br>By email<br><br>+ check box in the eCRF (Deviation table)	**Without delay** (Max 24 hours) **to the Sponsor**

5.3.5. Notification period to the sponsor

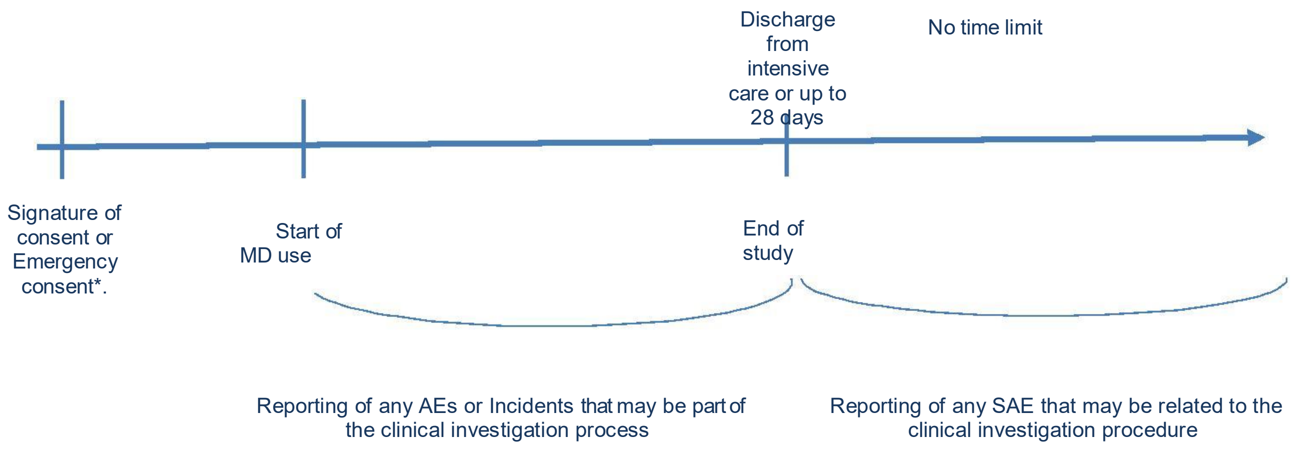

*Consent see details in § 7.8

The investigator is responsible for recording on the e-CRF and notifying the sponsor of the events listed in 5.3 of this protocol, occurring during the entire study:

- From 1ère administration of the study MD until discharge from the ICU or D28 if the patient is still in the ICU at D28.

Each event will be followed until complete resolution (stabilization at a level deemed acceptable by the investigator or return to the previous state), even if the patient is withdrawn from the study.

Special case - after completion of the study:

The sponsor should be informed if the investigator becomes aware of a serious adverse event that occurred after the end of the registration period and reporting of SAE period, without time limitation and without delay, when the SAE is likely to be related to the investigative procedure. These events must be reported using the Serious Adverse Event Reporting Form by sending paper forms by email to the following address: [redacted]

5.4. *ROLE OF THE SPONSOR*

5.4.1. Events to record

The sponsor records:

a) any adverse event defined in the protocol as critical to the evaluation of the study outcome

b) any serious adverse event related to the investigation procedure

c) any incident of a MD (only nature, start date, end date, severity, causality, action taken and evolution)
d) any new element concerning an event referred to in (a) to (c).
The sponsor is responsible for t h e ongoing evaluation of the safety of the investigative procedure.
The sponsor should verify the content, consistency of SAEs and assess:

- The causal relationship (unrelated, possible, probable, certain) between the serious adverse event and the investigative procedure,
- The expected or unanticipated nature of serious adverse events in the investigation procedure.

In the event of a different assessment by the sponsor and the investigator, both opinions are mentioned on the declaration sent to the competent authority if such a declaration is necessary.

Without prejudice to this centralized evaluation, the sponsor monitors the occurrence of MD-related complications in the context of its research via the AE/Incident table.

5.4.2. Declaration to the Competent Authority

The sponsor must report to the Competent Authority "**reportable events**"; **any serious adverse event** with a proven or reasonably foreseeable causal link (possible, probable, certain) with the investigation procedure, the new facts and, if applicable, the measures taken, as from the day on which it becomes aware of them. Only causality level 1 (i.e. "unrelated") is excluded from reporting. Deadlines for reporting by the sponsor to the ANSM :

Type of event	Time limit for initial declaration / Follow up
New fact	**WITHOUT DELAY**
A "reportable" event (SAE) that has resulted in death, imminent risk of death, serious injury or illness and requires prompt corrective action or new information on these cases.	**WITHOUT DELAY** and at the latest within **2 calendar days*.**

This includes relevant and unexpected events when they become a potential public health hazard. It also includes the possibility of multiple deaths occurring at short intervals.	
Other "deferrable" events and any new information relating to these events	**WITHOUT DELAY** and at the latest within **7 calendar days*.**

* from the moment of taking cognizance

5.5. *CONTACT VIGILANCE*

Center Hospitalier Départemental Vendée
Clinical Research Unit Boulevard
Stéphane MOREAU
85 925 LA ROCHE-SUR-YON Cedex 09
Tel : 02 51 44 65 72

5.6. INDEPENDENT SAFETY COMMITTEE (ISC)

The ISC is an advisory committee responsible for advising the sponsor and the study coordinator on the safety of a clinical trial. Its members are competent in the field of clinical trials (pathology, safety and ethics) and are not involved in the study. They are appointed for the remainder of the study and are committed to their participation as well as to respecting the confidentiality of the data. The choice of the members of the ISC is made in a collegial manner by the coordinator and the sponsor.
Within the framework of this study, a ISC is constituted after the start of the study following a New Safety Fact declaration transmitted to the ANSM on 22/11/2021.

Composition:

- Prof. Jean-Pierre QUENOT, Head of the Intensive Care Medicine Department - Dijon University Hospital and President of the CPP Est 1.
- Dr Jean Etienne HERBRECHT, Hospital practitioner - Intensive care unit - Intensive care unit - Hautepierre Hospital - Strasbourg.
- Mr Laurent POIROUX, PhD Nurse, Senior Health Executive, Paramedical Research Coordinator, Angers University Hospital, having worked for 14 years in intensive care.
- Pr Alexandre BOYER, Hospital practitioner - Intensive care unit - Resuscitation - Bordeaux University Hospital.
- Pr Mickael PIAGNERELLI, Hospital practitioner - Intensive medicine department - CHU of Charleroi, Belgium.

Role:

- Monitor the progress of the study, the characteristics of the patients included and the frequency of events of particular interest (self-extubations and repositioning of the endotracheal tube).
- Provide an advisory opinion on whether the trial should continue.
- The CIS is also notified of all security information transmitted to the appropriate authorities.

Operating procedures

The committee met in June 2022 and reported its findings before the beginning of the second period. In order to meet these deadlines, only the data from the first 300 included patients

were presented to the committee.
The committee decided to continue the study without any restrictions.
It may also meet at the request of the sponsor in the event of safety problems reported by the investigators during the study.
The committee shall inform the coordinator and the sponsor in writing of its findings.

The CIS will not have access to data concerning the primary endpoint (peri-oral lesions). No statistical tests will be performed, only descriptive data will be presented.
In case of a tendency to over-morbidity, a temporary suspension or a final decision of the research will be taken during a meeting organized by the coordinator, the different investigators and the members of the CIS.

6. DATA MANAGEMENT AND STATISTICS

6.1. COLLECTION AND PROCESSING OF STUDY DATA

6.1.1. Data collection

One electronic case report form (eCRF) will be created per patient. All information required by the protocol must be provided in the eCRF. It should include the data needed to confirm compliance with the protocol and all data needed for statistical analyses; it should identify major deviations from the protocol.

The person(s) responsible for completing the CRFs/eCRFs (investigator, CRA, etc.) must
/(s) must be defined and is/are identified in the delegation of responsibilities table for each center (kept in the investigator's binder).

6.1.2. Data coding

By signing this protocol, the principal investigator and all co-investigators agree to keep confidential the identities of the patients who participated in the study.

The transmission of a person's data for research purposes will therefore only be possible if a coding system is applied; the presentation of the research results must exclude any direct or indirect identification.

The identification of patients will be done according to the order of inclusion of patients by a number automatically assigned by the Clinsight software (eCRF) then completed by the initials of the patients (1st letter of the first name + 1st letter of the last name).

This code will be the only information that will appear on the eCRF notebook and will allow the eCRF to be linked to the patient afterwards.
The investigator is also required to code the patient data on all documents that he/she may have in his/her possession (reports of imaging or biological examinations, etc.) that are attached to the eCRF.

A correspondence table will be set up at the participating center. This table will be kept in a secure place by the principal investigator of the center and will contain the patient code and his nominative data in order to be able to go back to the patient file in case of missing or erroneous data. No clinical data will be collected in these correspondence tables.

6.1.3. Data processing

The collection of clinical data will be based on the implementation of a database and the creation of data entry masks similar to the observation book in accordance with the protocol and regulations currently in force.

6.2. STATISTICS

Lucie PLANCHE
CHD La Roche Sur Yon

The analyses will be carried out using the R software version 3.5.1

6.2.1. Description of methods statistics planned including schedule of planned interim analyses

The statistical analysis report will be written according to the recommendations proposed by Arnup et Al (13) on cross-over cluster trials.

Baseline features

A descriptive analysis of the clusters and patients will be presented for each of the 2 periods according to the sequence (cord fixation then adhesive strips // adhesive strips fixation then cord).

The description will include the numbers and percentages of terms for categorical variables and the minimum, maximum, mean, standard deviation and median for quantitative variables.

Analysis of the assessment criteria

To account for the study design, all criteria (primary and secondary) will be compared using hierarchical generalized linear models. The type of fixation, the period and the center (=cluster) will be taken into account in the model as fixed effects; the center*period interaction will be taken into account as a random effect (14).

Main criterion

The percentage of patients with at least one peri-oral lesion will be estimated with a 95% confidence interval for both types of fixation. A logistic regression model taking into account the duration of the patient's endotracheal tube retention and the study design will be applied.

Secondary criteria

The number of self-extubations, repositioning of the ETT will be estimated and compared using a Poisson regression model taking into account the study design. An offset of the logarithm of the intubation time will be included in the model.

The time to the first peri-oral lesion will be estimated using a random-effects survival model to account for the study design.

The time to onset will be calculated from the date of intubation to the date of first lesion onset. Patients for whom no lesion appeared will be censored at the date of extubation or at the date of discharge from intensive care or at D28. Patients who died will be censored at their date of death.

The grade of skin lesions (NPUAP scale) and mucosal lesions (Rompis scale) will be collected for each lesion. If the patient has no lesions, a grade=0 on the corresponding scale will be retained. If a patient presents several lesions, the maximum grade observed will be retained.
An ordinal logistic regression model controlling for intubation time and study design will be applied to compare both types of fixation.

The incidence of peri-oral lesions (expressed per 1000 ventilator days) will be calculated and described in each group: number of patients with at least one lesion / total number of ventilator days (up to 1ère lesions in patients with one lesion)

The maximum number of injuries per day will be estimated and compared using a linear model taking into account the study design.

6.2.2. Statistical justification of the number of inclusions

The study will take place in 16 centers (clusters) over two 12-month periods.

According to the IDEFIX study (7), we hypothesize that 35% of patients with a "cord and sleeve" fixation system have a peri-oral lesion compared to 20% with the elastic adhesive tape system.

The calculation of the number of subjects needed must take into account the design of the study and thus the two intra-class and inter-class correlation coefficients (15)

As we have no data on the estimation of correlation coefficients, we have retained, for the calculation of the number of subjects, a "moderate" intra-class correlation coefficient and therefore defined at 0.1, and as recommended, an inter-class correlation coefficient equal to half of the intra-class correlation coefficient, i.e. 0.05 (16)

With 90% power and 5% alpha risk, we calculated that each of the 16 centers would need to include an average of 24 patients per period.

A total of 768 patients are to be included.

6.2.3. Expected level of statistical significance

The alpha risk is set at 5%.

6.2.4. Statistical criteria for termination of research

NA

6.2.5. Method of accounting for missing, unused or invalid data

These are patients hospitalized in an intensive care unit. The risk of missing data is a priori very low.

In all cases, the missing data will be presented for each of the 2 periods according to the sequence (cord fixation system then adhesive strips // adhesive strips fixation system then cord). The reasons for the missing data will be researched and also presented.

For the analysis of the main criterion (appearance of a lesion), in case of missing data on the expert assessment on photo, we will impute this data by the nurse's assessment. A sensitivity analysis will be applied on the imputation of missing data by considering that the patient did not present a lesion (whatever the period and the type of fixation used).

6.2.6. Managing changes to the initial strategy analysis plan

NA

6.2.7. Selection of individuals to be included in the analyses

A flowChart as proposed by Arnup et al (13) will be presented to describe the participation of the centers for each of the 2 periods.

The main analysis will be performed on the Intent-to-Treat (ITT) population, i.e. on all randomized patients.

A Per-Protocol analysis will also be presented including all patients for whom no major deviation from the protocol was noted.
A data review meeting will be held to review and define the major criterion or not for each of the deviations.

6.2.8. Randomization

This is a study with a cluster cross-over design.

The study will take place over 2 periods of 12 months.
In the first period, 8 centers will use the corded fastening system and the other 8 centers will use the tape fastening system. In the second period, each of these centers will use the other fastening system.
The randomization will therefore consist in defining for each of these 16 centers in which order the fixation system will be used.

The randomization will be carried out before the implementation of the study and will be performed by the statistician of the Research Unit of the CHD of La Roche sur Yon.

7. ADMINISTRATIVE AND REGULATORY ASPECTS

7.1. *RIGHT OF ACCESS TO SOURCE DATA AND DOCUMENTS*

In accordance with GCP:

- The sponsor is responsible for obtaining the agreement of all parties involved in the research to ensure direct access to all research sites, source data and reports for quality control and auditing purposes by the sponsor,
- The investigators will make available to the persons in charge of monitoring, quality control or audit of the biomedical research, the documents and individual data strictly necessary for this control, in accordance with the legislative and regulatory provisions in force (articles L.1121-3 and R.5121-13 of the public health code).

Source documents are defined as any original document or object that can be used to prove the existence or accuracy of a data or fact recorded during the clinical study and will be kept for 15 years by the investigator or by the hospital in the case of a hospital medical record.

7.2. *DATA PRIVACY*

Persons having direct access will take all necessary precautions to ensure the confidentiality of information relating to the persons who have access, particularly with regard to their identity and the results obtained.

These persons, as well as the investigators themselves, are subject to professional secrecy (according to the conditions defined by articles 226-13 and 226-14 of the penal code).

During or at the conclusion of the research, the data collected on the individuals involved in the research and provided by the researchers will be anonymized.

Under no circumstances should the names of the persons concerned or their addresses appear in clear text.

Only the first letter of the subject's name and the first letter of the subject's first name will be recorded, along with a coded number specific to the study indicating the order of inclusion of the subjects.

7.3. MONITORING OF THE TRIAL

Monitoring will be carried out by the Promotion Department of the Research Directorate. A Clinical Research Associate (CRA) will regularly visit each site (investigator and pharmacy) to check the quality of the data reported in the observation books.

The monitoring plan is defined in consultation between the research team and the responsible institution according to the objectives of the study.

The protocol has been classified according to the estimated level of risk to the patient undergoing the research. It will be followed in the following manner:

Risk A: low or negligible foreseeable risk

On-site monitoring visits will be arranged after an appointment with the investigator. The CRAs will have access to each site:
- the data collection books of the included patients,
- patient medical and nursing records,
- the investigator binder.

7.4. INSPECTION / AUDIT

As part of this study, an inspection or audit may take place. The sponsor and/or participating centers must be able to provide access to the data to the inspectors or auditors.

7.5. DECLARATION TO THE COMPETENT AUTHORITIES

The sponsor undertakes to submit the study project for prior authorization by a Committee for the Protection of Persons (CPP). The information communicated concerns, on the one hand, the modalities and nature of the research and, on the other hand, the guarantees provided for patients participating in this trial.

This protocol will also be reported to the ANSM.

7.6. *AMENDMENTS TO THE PROTOCOL*

Requests for substantial modifications will be sent by the sponsor to the CPP concerned for its opinion and to the ANSM for information, in accordance with the law in force and its implementing decrees.

The modified protocol will have to be the subject of an updated dated version.
The patient information and consent forms will need to be modified if necessary.

7.7. *COMPUTERIZED DATA AND SUBMISSION TO THE CNIL*

The data collected in this study is for scientific research purposes, for the public good.

This study falls within the framework of the "Reference Methodology" MR-001 registered, for the CHD Vendée, under n°2060482 v 0 for the following reasons
- Collection of health data for research purposes
- Obtaining the opinion of a CPP to start the research
- Use of anonymized data
- Individual information of the persons concerned
- Access to data only by professionals (health care and sponsor) involved in the study.

The fact that this study falls within the scope of MR001 and the reasons for it will be notified in the sponsor's treatment registry.

7.8. *PATIENT INFORMATION*

7.8.1. Oral informed consent

The investigator (in the context of this study: intensive care nurse or intensive care physician) undertakes to obtain the free, informed and express consent of the patient if he/she is capable, or of the trusted person or, failing that, of one of his/her relatives if the patient is not capable, after having provided him/her with information about the protocol (information note and form for collecting

consent form in Appendix 3, 4, 5). He will give him a copy of the information note and an oral consent form. The person can only be included in the study after having read the information note and given oral consent after having had time to reflect, if necessary.

If the information was initially given to the patient's family, the patient will be informed as soon as possible and asked for consent to continue the research if he or she regains the capacity to consent. A copy of the information note will also be given to the patient.

As a last resort and in the physical absence of a trusted person or loved one at the time of inclusion, an emergency procedure will be available to include the patient.
In all cases, the family member and then the patient will be informed as soon as possible by the investigator, the information note will be delivered to them and the collection of their oral consent will be carried out secondarily by the investigator, or a physician representing him.

The information of the patient or of the trusted person or, failing that, of one of his relatives and his agreement to participate in the research must be noted in his medical file as well as his potential inclusion in the emergency.

Patients under guardianship should not normally be included (exclusion criteria). The patient often lacks the required level of consciousness at the time of inclusion. The collection of consent is often done with relatives.
Sometimes the discovery of the guardianship of the patient is made after randomization and consent of a relative. These patients will be excluded from the study and their data will not be included in the analysis.

7.8.2. Rationale for requesting patient inclusion under an emergency protocol

This research falls within the framework of article L. 1122-1-3 of the public health code. Indeed, the inclusion criteria imply the inclusion of patients requiring mechanical ventilation via intubation. The patient will therefore not be in a position to give his consent to participate in the study. In accordance with Article L. 1122-1-3 of the Public Health Code, the consent of this person will not be sought and only that of the members of his family or that of the trusted person mentioned in Article L. 1111-6 under the conditions provided for in Article L. 1122-1-1, if they are present, will be sought.

In this cluster study, patients will have the fixation system present in the department whether or not they are included in the study, so the risks and constraints of inclusion in the study are minimal. The time frame for patient inclusion is quite short. The patient must be included before the second photo taken on Day 1 when the fixation is changed. The relatives sometimes do not have the time to come to the department in this time interval. Thus, if the trusted person or a relative has not come to the department during this period and the information note has not been delivered and the verbal consent of the relative has not been obtained, an emergency procedure requesting the investigating physician will be implemented. The information and the absence of opposition of the trusted person or a close relative and then of the patient will be collected as soon as possible.

7.9. FINANCING AND INSURANCE

The Sponsor ensures the financing of the study and subscribes to an insurance policy guaranteeing the pecuniary consequences of its civil liability, in accordance with the regulations.

7.10. RULES FOR PUBLICATION

The study will be registered on an open access website (Clinical trial) before the inclusion of the first patient in this study.

The scientific communications and reports corresponding to this study will be carried out under the responsibility of the study coordinator with the agreement of the principal investigators of the participating centers.

The coordinating investigator establishes the list of authors.
A physician or paramedical staff member attached to the CHD Vendée at the time of publication will necessarily be listed as the first or last author.

The rules of publication will follow international recommendations (N Engl J Med, 1997; 336:309-315).

A copy of the publication will be given to the CHD Vendée Sponsor of the study, which will necessarily be cited

7.11. *ARCHIVING OF SOURCE DATA*

The investigator must retain all study information for at least 15 years after the end of the study. At the end of the study, the investigator will receive a copy of each patient's data from the sponsor.

No removal or destruction shall be made without the consent of the Developer. At the end of the 15 years, the Developer will be consulted for destruction. All data, documents and reports may be subject to audit or inspection.

8. REFERENCES

1. Vangilder C, Amlung S, Harrison P, Meyer S. Results of the 2008–2009 International Pressure Ulcer Prevalence™ Survey and a 3-Year, acute care, unit-specific analysis. Ostomy Wound Manage. 1 nov 2009;55:39-45.

2. A Prospective, Descriptive Study to Determine the Rate and Characteristics of and Risk Factors for the Development of Medical Device-related Pressure Ulcers in Intensive Care Units [Internet]. Wound Management & Prevention. 2016 [cité 12 mars 2019]. Disponible sur: https://www.o-wm.com/article/prospective-descriptive-study-determine-rate-and-characteristics-and-risk-factors

3. qrg_prevention_in_french.pdf [Internet]. [cité 4 sept 2019]. Disponible sur: http://www.epuap.org/wp-content/uploads/2016/10/qrg_prevention_in_french.pdf

4. A Prospective, Descriptive Study to Assess Nursing Staff Perceptions of and Interventions to Prevent Medical Device-related Pressure Injury [Internet]. Wound Management & Prevention. [cité 21 août 2019]. Disponible sur: https://www.o-wm.com/article/prospective-descriptive-study-assess-nursing-staff-perceptions-and-interventions-prevent

5. Widiati E, Nurhaeni N, Gayatri D. Medical-Device Related Pressure Injuries to Children in the Intensive Care Unit. Compr Child Adolesc Nurs. 30 nov 2017;40(sup1):69-77.

6. Cooper K. Evidence-Based Prevention of Pressure Ulcers in the Intensive Care Unit [Internet]. 2013 [cité 12 mars 2019]. Disponible sur: http://ccn.aacnjournals.org/content/33/6/57.long

7. Societe de Reanimation de Langue Francaise. 20111001-CERC-Abstract_RIR_IDEFIX_1_Congres_2012-abstract-au-congres-srlf-2012.pdf [Internet]. SRLF. 2011 [cité 8 mars 2019]. Disponible sur: https://www.srlf.org/wp-content/uploads/2015/12/20111001-CERC-Abstract_RIR_IDEFIX_1_Congres_2012-abstract-au-congres-srlf-2012.pdf Navigateur : internet explorer

8. Kim C-H, Kim MS, Kang MJ, Kim HH, Park NJ, Jung HK. Oral mucosa pressure ulcers in intensive care unit patients: A preliminary observational study of incidence and risk factors. J Tissue Viability. févr 2019;28(1):27-34.

9. Reaper S, Green C, Gupta S, Tiruvoipati R. Inter-rater reliability of the Reaper Oral Mucosa Pressure Injury Scale (ROMPIS): A novel scale for the assessment of the severity of pressure injuries to the mouth and oral mucosa. Aust Crit Care. mai 2017;30(3):167-71.

10. Fisher DF, Chenelle CT, Marchese AD, Kratohvil JP, Kacmarek RM. Comparison of commercial and noncommercial endotracheal tube-securing devices. Respir Care. sept 2014;59(9):1315-23.

11. Hampson J, Green C, Stewart J, Armitstead L, Degan G, Aubrey A, et al. Impact of the introduction of an endotracheal tube attachment device on the incidence and severity of oral pressure injuries in the intensive care unit: a retrospective observational study. BMC Nurs [Internet]. déc 2018 [cité 1 févr 2019];17(1). Disponible sur: https://bmcnurs.biomedcentral.com/articles/10.1186/s12912-018-0274-2

12. for the Pragmatic Critical Care Research Group, Landsperger JS, Byram JM, Lloyd BD, Rice TW. The effect of adhesive tape versus endotracheal tube fastener in critically ill adults: the

endotracheal tube securement (ETTS) randomized controlled trial. Crit Care. déc 2019;23(1):161.

13. Arnup SJ, Forbes AB, Kahan BC, Morgan KE, McKenzie JE. The quality of reporting in cluster randomised crossover trials: proposal for reporting items and an assessment of reporting quality. Trials. déc 2016;17(1):575.

14. Morgan KE, Forbes AB, Keogh RH, Jairath V, Kahan BC. Choosing appropriate analysis methods for cluster randomised cross-over trials with a binary outcome: K. E. MORGAN *ET AL.* Stat Med. 30 janv 2017;36(2):318-33.

15. Giraudeau B, Ravaud P, Donner A. Sample size calculation for cluster randomized cross-over trials. Stat Med. 29 nov 2008;27(27):5578-85.

16. Sample size calculations for stepped wedge and cluster randomised trials: a unified approach - ScienceDirect [Internet]. [cité 10 sept 2019]. Disponible sur: https://www.sciencedirect.com/science/article/pii/S089543561500414X

17. Boulain T. Unplanned extubations in the adult intensive care unit: a prospective multicenter study. Association des Reanimateurs du Centre-Ouest. Am J Respir Crit Care Med 1998;157(4 Pt 1):1131-1137.
