# Supplementary material for: Impact of two endotracheal tube fixation on the incidence of peri-oral lesions: Elastic adhesive strips versus cord in a protective sheath. Study protocol for a cluster cross-over randomized trial
Source: PLoS One. 2024 Feb 8;19(2):e0297349. doi: 10.1371/journal.pone.0297349 (PMC10852271; doi:10.1371/journal.pone.0297349)
Supplement: S1 Appendix — (PDF) [file pone.0297349.s003.pdf]

Submission: SRLF 2012 Congress

Injuries associated with intubation tube fixation: sheath or adhesive? IDEFIX study

C Commission for Epidemiology and Clinical Research (1);
(1) SRLF, Société de Réanimation de Langue Française, Paris.

Abstract number : **005354 (FR)**
Speaker: **C Commission for Epidemiology and Clinical Research**
Structure : **(Nursing convention) Nurse**
References :

Study mode: **Clinical study**
Certificate of conformity:
CCPPRB certificate: **No**
Ethics Committee certificate: **Yes**
Commitment to transfer rights: **Yes**
Done on **06/10/2011**

Introduction

Fixation of intubation tubes is common practice in the intensive care unit. To the best of our knowledge, no studies have been carried out on mucocutaneous lesions induced by different types of fixation. The aim of this study was to assess the prevalence of these lesions and identify their risk factors.

Patients and Methods

Multicentric (20 centers), French-speaking, prospective, observational 2-month study carried out by nurses. Patients hospitalized in intensive care, intubated/ventilated for less than 12 hours were included. Lesions (according to the pressure sore classification) induced by the fixation system were monitored daily until extubation, or for a maximum of 30 days. The type of fixation (adhesive, sheath, cord) as well as general risk factors (SAPS II, presence or absence of amines, agitation, transport) and local risk factors (Guédel, poor oral condition) were also collected. Results are expressed as median [IQR 25-75].fixation types were compared using a chi-square test.

Results

448 patients were included (11 newborns; 437 adults : SAPS II: 52 [38-68], 49 % on catecholamines, 33% transported on D1, mortality on D28: 36%, duration of ventilation: 5 days [2-8]). 385 patients were analyzed (52 data could not be analyzed). Three types of fixation were used: cord 41%, sheath 38%, adhesive 21%. 141 patients (36%) developed lesions. The proportion of lesions was 23% at D1, 36% at D3 and 53% at D5. They were significantly less frequent with adhesive fixation (cord 43%, sheath 43%, adhesive 13%; p<0,001).

Conclusion

More than a third of patients had lesions associated with intubation tube attachment. Tights seem to induce fewer lesions, but this needs to be confirmed in other studies.
